# Outcomes for ER-positive *CHEK2* c.1100delC breast cancer patients compared with breast cancer patients without the variant

**DOI:** 10.1016/j.breast.2025.104666

**Published:** 2025-11-24

**Authors:** Maartje A.C. Schreurs, Muriel A. Adank, Bernadette A.M. Heemskerk-Gerritsen, Antoinette Hollestelle, Nyrée Smallenbroek, Christi J. van Asperen, Margreet G.E.M. Ausems, Irma van de Beek, Geertruida H. de Bock, Ingrid Boere, Liselotte P. van Hest, Kim J.A.F. van Kaam, Linda de Munck, Janet R. Vos, Agnes Jager, Marjanka K. Schmidt, Maartje J. Hooning, Hebon

**Affiliations:** a Department of Medical Oncology, Erasmus MC Cancer Institute, Rotterdam, the Netherlands; b Department of Clinical Genetics, Netherlands Cancer Institute, Amsterdam, the Netherlands; c Department of Clinical Genetics, Leiden University Medical Center, Leiden, the Netherlands; d Division of Laboratories, Pharmacy and Biomedical Genetics, Department of Genetics, University Medical Center Utrecht, Utrecht, the Netherlands; e Department of Epidemiology, University of Groningen, University Medical Center Groningen, Groningen, the Netherlands; f Department of Human Genetics, Section Clinical Genetics, Amsterdam UMC, University of Amsterdam, Amsterdam, the Netherlands; g Department of Clinical Genetics, Maastricht University Medical Center, Maastricht, the Netherlands; h Department of Research and Development, Netherlands Comprehensive Cancer Organisation, Utrecht, the Netherlands; i Department of Human Genetics, Radboud University Medical Center, Nijmegen, the Netherlands; j Division of Psychosocial Research and Epidemiology, Netherlands Cancer Institute, Amsterdam, the Netherlands; k Division of Molecular Pathology, Netherlands Cancer Institute, Amsterdam, the Netherlands; l Department of Clinical Genetics, Erasmus MC Cancer Institute, Rotterdam, the Netherlands

**Keywords:** CHEK2 c.1100delC, Breast cancer outcomes, ER-Positive breast cancer, Prognosis

## Abstract

**Purpose:**

Germline *CHEK2* c.1100delC-associated breast cancer (BC) patients have been reported with worse prognosis than patients without the variant. However, results are based on older cohorts and treatment regimens. As part of the Hebon-CHEK2 study, we aim to study prognosis in a Dutch cohort of genetically tested ER-positive BC patients diagnosed from 2006 onwards.

**Methods:**

All patients underwent genetic testing based on personal and family history risk, and data on BC outcomes were collected. Hazard ratios (HRs) and 95 % confidence intervals (CI) for the association of *CHEK2*-status with prognosis were estimated via delayed entry Cox regression models, adjusted for age and year of diagnosis, tumor size, nodal status, and primary treatment regimens. Furthermore, we meta-analyzed our results with previous studies.

**Results:**

We included 480 *CHEK2* BC patients and 944 BC patients without the variant. Median follow-up was 6.0 years. Heterozygotes were more often diagnosed with small tumors, and lymph node positive disease. No significant difference was found for recurrent disease and distant disease-free survival, neither before 5 years (HR = 0.73; 95 %CI = 0.35–1.53 and HR = 0.99; 95 %CI = 0.44–2.21, respectively), nor after 5 years follow-up (HR = 0.29; 95 %CI = 0.06–1.28 and HR = 0.39; 95 %CI = 0.10–1.39, respectively). Also no significant difference in BC-specific survival (HR = 0.77; 95 %CI = 0.42–1.39) or overall survival (HR = 0.69; 95 %CI = 0.43–1.08) was found. Meta-analysis of our results with previous studies showed a worse BC-specific survival for heterozygotes.

**Conclusion:**

In our study, with more recent years of diagnosis and treatment, we found no difference in prognosis, as opposed to previous studies. Further research is needed to validate our findings.

## Highlights

• Previous studies consistently showed a worse prognosis in *CHEK2* c.1100delC patients.
• Current study includes modern treatment regimens representing current patients.
• Here, no difference in prognosis for patients diagnosed from 2006 onwards was found.
• However, our meta-analysis with older data still showed a worse BC-specific survival.

## 1 Introduction

The pathogenic germline *CHEK2* c.1100delC variant is commonly found in the Netherlands; 1 % of the general population is heterozygous for *CHEK2* c.1100delC [1]. *CHEK2* c.1100delC heterozygotes have an increased risk of breast cancer and contralateral breast cancer [[2], [3], [4], [5]]. Also, they are diagnosed at a younger average age than sporadic breast cancer patients [6]. Although heterozygotes are predominantly diagnosed with hormone receptor positive tumors [[7], [8], [9]], there is evidence that the tumors have different molecular characteristics compared to estrogen receptor (ER)-positive tumors in patients without a *CHEK2* c.1100delC variant (from here onward referred to as “non-carriers”) [10], which could result in less favorable response to treatment. Previous studies have consistently reported a worse prognosis for *CHEK2* c.1100delC breast cancer patients [3,5,6,11] and a higher incidence of recurrent disease and distant metastatic disease than non-carriers [7,8,12]. However, so far, there is no evidence that heterozygotes respond differently to breast cancer treatment than non-carriers [5]. In the Netherlands, diagnostic testing for *CHEK2* c.1100delC was implemented in September 2014 [13].

While there is a need for prospective data collection to answer questions on prognosis after breast cancer occurrence in *CHEK2* c.1100delC heterozygotes [14], a first step would be to study the prognosis in a recent cohort. The last two decades, many novel treatment regimens have become available which have improved prognosis. For example, the introduction of anti-human epidermal growth factor receptor 2 (HER2)-targeted therapy around 2006 significantly increased survival of HER2-positive breast cancer patients [15]. Since then, the use of aromatase inhibitors compared with tamoxifen in ER-positive breast cancer has significantly improved prognosis in postmenopausal women [16,17]. Also in more recent years, CDK4/6 inhibitors in combination with endocrine therapy have further improved prognosis [18,19].

We selected all breast cancer patients who were identified within the Hebon-CHEK2 study [20], a nationwide study on hereditary breast and ovarian cancer with a special focus on *CHEK2* c.1100delC families. We focused on ER-positive breast cancer patients diagnosed from 2006 onwards. This provides the unique opportunity to study overall survival, breast cancer-specific survival, distant disease-free survival and recurrent disease-free survival in a large nationwide setting, taking current treatment regimens into account. We also meta-analyzed the results from previous studies and our own on prognosis in *CHEK2* c.1100delC-associated breast cancer patients.

## 2 Methods

### 2.1 Study population

Recently, women from *CHEK2* c.1100delC families who underwent genetic testing were invited to participate in the nationwide Hereditary Breast and Ovarian cancer (Hebon) study [20]. When also other breast cancer-associated pathogenic variants had been tested, we checked if these results were negative, to ensure that we purely study the impact of the *CHEK2* c.1100delC variant. In short, we included 1,802 women from *CHEK2* c.1100delC families, including responders, non-responders and women who were deceased at the time of invitation. After linkage with the Netherlands Cancer Registry, through a third trusted party, we received pseudonymized data on age at and year of breast cancer diagnosis, tumor characteristics, treatments given and vital status. Additional information on the moment of recurrent or metastatic disease and the location of the disease was collected by the Netherlands Cancer Registry to assess recurrent disease, metastatic disease and breast cancer-specific survival for the responders and the women who deceased before the invites were sent out, as exclusion of the deceased women would introduce bias (paragraph 5.4.2.b. of the Dutch Code of Conduct for Health Research). However, this additional information was not available for the non-responders, as this was not possible according to the General Data Protection Regulation.

**Fig. 1:**
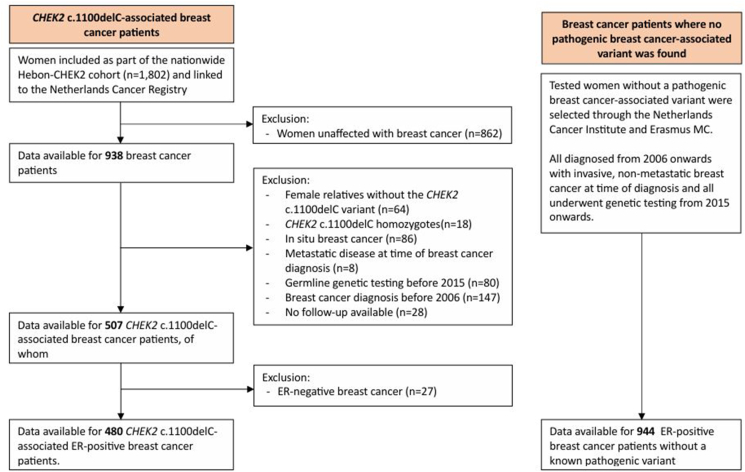
Flowchart of the patients included in this study. Pseudonymized data was retrieved from two parallel selection strategies, and therefore it was not possible to link patients on an individual level. As the number of genes included in the panels increased over time, and part of the women underwent targeted testing for one particular familial pathogenic variant, there might still be some carriers for other pathogenic variants present within the current study population.

In this study, we included heterozygotes diagnosed with invasive, non-metastatic, ER-positive breast cancer. As comparison group, tested non-carrier patients with ER-positive breast cancer diagnosed in the same period from two hospitals that already provided heterozygotes were added to the cohort (Fig. 1). All these patients underwent genetic testing for at least *BRCA1/2* and *CHEK2* 1100delC, and no pathogenic variants have been found. Similar to the heterozygotes, clinical information and follow-up was collected from the hospitals. All patients underwent genetic testing from 2015 onwards. Based on the current Dutch guidelines, there are no differences in screening regimens between the groups.

### 2.2 Statistical analyses

Statistical analyses were performed using STATA (version 15.0). Descriptive statistics are shown as mean ± standard deviation (SD) or median (range). We used Pearson's χ2 test and Fisher's exact test for categorical data and *t*-test for continuous data to calculate differences in patients' characteristics. A two-tailed test was used to determine whether the differences were significant.

#### 2.2.1 Survival analyses

For the survival analyses, we considered age and year of breast cancer diagnosis, nodal status and tumor size as potential confounders, based on current knowledge [[2], [3], [4], [5], [6],^11^]. Furthermore, we included radiotherapy, endocrine therapy, chemotherapy and targeted therapy given as part of the primary treatment as variables of interest, as we aimed to study the impact on prognosis, taking the current treatment regimens into account. Hazard ratios (HRs) and 95 % confidence intervals (95 %CI) for the association of *CHEK2* c.1100delC status with time to event were estimated via delayed entry (left-truncated) Cox regression models. For the overall survival analyses, time-to-event started at first primary breast cancer diagnosis or time of genetic testing, whichever came last, and ended at time of death. Patients were censored at last available follow-up (i.e. linkage with the Dutch Personal Records database to obtain vital status), or 10 years after first primary breast cancer diagnosis, whichever came first. Cause of death was used to determine breast cancer-specific survival. If cause of death was unknown, but the patient was previously diagnosed with distant disease, we assumed breast cancer as cause of death [21].

Since no information on cause of death or distant disease was provided for non-responders, we performed several sensitivity analyses to understand the potential impact of non-responder data on our analyses on breast cancer-specific survival. First, we included all seven deceased non-responders with cause of death coded as breast cancer as these patients were relatively young at time of breast cancer diagnosis (five out of the seven non-responders were diagnosed before the age of 45) and therefore unlikely that they died due to other causes. Second, we also did the analysis with cause of death for the deceased non-responders coded as death due to other cause. Third, we excluded the data from all 117 non-responders to determine the impact of using non-responder data in this analysis.

For the recurrent disease-free survival analyses, time-to-event started at first primary breast cancer diagnosis or time of genetic testing, whichever came last, and ended at occurrence of loco-regional recurrence or metastatic disease. For the distant disease-free survival analyses, time-to-event was until occurrence of metastatic disease. Patients were censored at last available follow-up (last information from hospital), or 10 years after first breast cancer diagnosis, whichever came first. Follow-up was cut after 10 years as there was too little power to study these outcomes after that time. Last information from the hospital was used to ensure that no recurrent disease was missed. Since information on loco-regional recurrence and distant disease was not available for the *CHEK2* c.1100delC non-responders, they were therefore excluded from these specific analyses resulting in smaller sample size to study the association.

Additional analyses were performed by tumor subtype: (1) ER-positive/HER2-negative breast cancer patients; and (2) ER-positive/HER2-positive breast cancer patients. As Trastuzumab is not prescribed for HER2-negative breast cancer patients, this variable was excluded in the corresponding analysis.

**Fig. 2:**
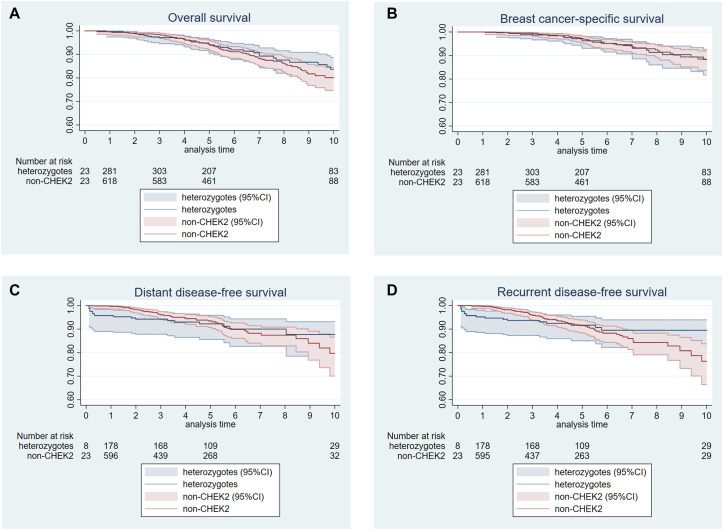
Survival curves representing the (A) overall survival, (B) breast cancer-specific survival, (C) distant disease-free survival and (D) recurrent disease-free survival up to 10 years after breast cancer diagnosis among ER-positive *CHEK2* c.1100delC breast cancer patients (blue line) and breast cancer patients without the variant (red line). (For interpretation of the references to colour in this figure legend, the reader is referred to the Web version of this article.)

The proportional hazards assumption was assessed by using an extended Cox model with time-depending covariables. The extended models for recurrent and distant disease-free survival violated the proportional hazards assumption (p-value = 0.03 and p-value = 0.04, respectively), indicating that the hazards were not proportional over time. Because the curves for distant disease-free survival and recurrent disease-free survival for *CHEK2* heterozygotes and non-carriers started to diverge after 5 years (Fig. 2C and D), we chose for an extended Cox model with a cut-off point at the time of diversion. The corresponding models then provided two hazard ratios for *CHEK2* 1100delC, one for the first part and one for the latter part.

#### 2.2.2 Meta-analysis

A literature search was conducted to identify all relevant publications on prognosis in *CHEK2* c.1100delC breast cancer patients. As the main majority of *CHEK2* c.1100delC women is diagnosed with ER-positive breast cancer, we decided to include all studies published on prognosis in *CHEK2* c.1100delC breast cancer patients, rather than limiting to ER-positive breast cancer only. We extracted data on number of patients included, number of heterozygotes, age at diagnosis, year of diagnosis, methodology used in the data analysis, and outcomes from all relevant publications. Furthermore, we evaluated the potential overlap in heterozygotes between the studies and combined the results in a meta-analysis.

The relative risk estimates with the corresponding 95 %CIs from the multivariable analyses were collected, log-transformed and meta-analyzed per outcome. Both a random-effects model and a fixed-effects model with an inverse variance-weighted method were used for the meta-analysis [22]. In addition, the heterogeneity between studies was reported to determine the best suited model for each analysis.

## 3 Results

In total, 480 heterozygotes and 944 non-carriers were included with ER-positive breast cancer (Fig. 1, Table 1). The median time of follow-up was 6 years. Furthermore, *CHEK2* c.1100delC breast cancer patients were diagnosed with smaller tumors (67.3 % vs 55.0 % of tumors ≤2 cm) but more often lymph node positive disease (44.3 % vs 30.9) compared to non-carriers. Also, the tumors were more often HER2-positive in *CHEK2* c.1100delC patients than non-carriers (22.6 % vs. 17.3 %).

**Table 1:** Patient-, tumor- and treatment characteristics of women diagnosed with ER-positive breast cancer included in the analyses.

	*CHEK2* c.1100delC heterozygotes (n = 480)	non-*CHEK2(* n = 944)	*P*-value
**Patient characteristics**			
Age at first diagnosis (years), mean (SD)	48.2 (11.0)	47.7 (10.9)	0.41^a^
Age categories, n (%)			0.53^b^
<40	101 (21.0)	217 (23.0)	
40-49	170 (35.4)	351 (37.2)	
50-59	133 (27.7)	230 (24.4)	
≥60	76 (15.8)	146 (15.5)	
Year at first diagnosis, median (range)	2015 (2006–2021)	2016 (2006–2023)	<0.001^a^
Year categories, n (%)			<0.001^b^
<2010	40 (8.3)	33 (3.5)	
2010–2014	136 (28.3)	152 (16.1)	
2015–2019	296 (61.7)	572 (60.6)	
≥2020	8 (1.7)	187 (19.8)	
Follow-up time from breast cancer diagnosis			
Mean (SD)	6.5 (3.1)	6.0 (3.3)	0.001^a^
Median (range)	5.9 (0.9–15.8)	6.3 (0.2–16.6)	0.04^c^
**Tumor characteristics**			
Tumor size, n (%)			<0.001^b^
≤2 cm	323 (67.3)	519 (55.0)	
>2 and ≤ 5 cm	119 (24.8)	341 (36.1)	
>5 cm	38 (7.9)	84 (8.9)	
Nodal status, n (%)			<0.001^b^
Negative	267 (55.7)	652 (69.1)	
Positive	212 (44.3)	292 (30.9)	
Missing, n	1	0	
Grade			0.68^b^
1	91 (20.8)	171 (19.1)	
2	240 (54.8)	511 (57.2)	
3	107 (24.4)	212 (23.7)	
Missing, n	42	50	
Morphology, n (%)			<0.01^d^
NST	399 (83.1)	720 (76.3)	
Lobular	29 (6.0)	111 (11.8)	
Medullary	1 (0.2)	1 (0.1)	
Mixed (ductal & lobular)	17 (3.5)	50 (5.3)	
Mucinous	8 (1.7)	11 (1.2)	
Tubular	3 (0.6)	3 (0.3)	
Other	23 (4.8)	48 (5.1)	
PR status, n (%)			0.16^b^
Negative	76 (15.9)	179 (19.0)	
Positive	401 (84.1)	764 (81.0)	
Missing, n	3	1	
HER2 status, n (%)			0.05^d^
Negative	367 (76.9)	774 (82.3)	
Positive	108 (22.6)	163 (17.3)	
Equivocal	2 (0.4)	4 (0.4)	
Missing, n	3	3	
**Treatment**			
Surgery, n (%)			0.30 ^b^
Breast conserving surgery	244 (50.8)	510 (54.0)	
Mastectomy	236 (49.2)	432 (45.8)	
Type unknown	0	2 (0.2)	
Radiotherapy, n (%)			<0.001 ^b^
No	173 (36.0)	169 (17.9)	
Yes	307 (64.0)	775 (82.1)	
Endocrine therapy, n (%)			0.20 ^b^
No	101 (21.0)	227 (24.1)	
Yes	379 (79.0)	717 (76.0)	
Of whom, type endocrine therapy, n (%)			<0.001 ^b^
Tamoxifen	210 (55.4)	247 (34.5)	
Aromatase inhibitors	52 (13.7)	88 (12.3)	
Other, unknown type	117 (30.9)	382 (53.3)	
Chemotherapy, n (%)			0.47 ^b^
No	166 (34.6)	345 (36.6)	
Yes	314 (65.4)	599 (63.5)	
Trastuzumab, n (%)			0.13 ^b^
No	376 (78.3)	771 (81.7)	
Yes	104 (21.7)	173 (18.3)	

CBC = contralateral breast cancer; ER = estrogen receptor; HER2 = human epidermal growth factor receptor 2; PR = progesterone receptor. Percentages might not add up to 100 %, due to rounding.

P-values were determined by different statistical tests. ^a^: *t*-test; ^b^: Chi-square test, ^c^: Mood's median test, ^d^: Fisher's exact test.

### 3.1 Survival analyses

Survival curves are presented to visualize the overall survival (Fig. 2A, log-rank test p-value = 0.17), breast cancer-specific survival (Fig. 2B, log-rank test p-value = 0.88), distant disease-free survival (Fig. 2C, log-rank test p-value = 0.32) and recurrent disease-free survival (Fig. 2D, log-rank test p-value = 0.12) in ER-positive breast cancer patients. The univariable analysis (Supplementary Tables 1–2) showed no significant difference in overall survival, breast cancer-specific survival, distant disease-free survival and recurrent disease-free survival between heterozygotes and non-carriers. After adjustment for potential confounders (Table 2), again, *CHEK2* c.1100delC status was not significantly associated with differences in overall survival (HR = 0.69; 95 %CI = 0.43–1.08) and BC-specific survival (HR = 0.77; 95 %CI = 0.42–1.39). Also, no significant differences in distant disease-free survival and recurrent disease-free survival was found, neither before 5 years (HR = 0.99; 95 %CI = 0.44–2.21 and HR = 0.73; 95 %CI = 0.35–1.53, respectively), nor after 5 years follow-up (HR = 0.39; 95 %CI = 0.10–1.39, and HR = 0.29; 95 %CI = 0.06–1.28, respectively). The additional analyses did not change the conclusions (Supplementary Tables 4–6).

**Table 2:** Multivariable analyses of association between *CHEK2* c.1100delC and breast cancer-specific survival and overall survival (hazard ratio) in ER-positive breast cancer patients.

	Breast cancer-specific survival		Overall survival	
	*CHEK2* heterozygotes	non-*CHEK2*	*CHEK2* heterozygotes	non-*CHEK2*
Patients, n	479	943	479	943
Events, n	19	39	30	74
PYO	1959	4061	1959	4061
Incidence rate/1000 PYO	9.7	9.6	15.3	18.2
	**HR (95** **%CI)**	**p-value**	**HR (95** **%CI)**	**p-value**
*CHEK2* c.110delC status				
Non-carrier	ref.		ref.	
Heterozygotes	0.77 (0.42–1.39)	0.38	0.69 (0.43–1.08)	0.11
Age at diagnosis (per year increase)	0.98 (0.96–1.01)	0.28	1.02 (1.00–1.04)	0.10
Year at diagnosis (per year increase)	0.91 (0.78–1.06)	0.23	0.91 (0.81–1.03)	0.13
Tumor size				
<2 cm	ref.		ref.	
2–5 cm	1.05 (0.58–1.92)	0.87	1.53 (0.96–2.43)	0.07
>5 cm	1.95 (0.93–4.06)	0.08	3.65 (2.12–6.29)	<0.001
Nodal status				
Negative	ref.		ref.	
Positive	3.65 (1.91–6.95)	<0.001	2.18 (1.38–3.43)	0.001
Endocrine therapy				
None	ref.		ref.	
Tamoxifen	1.75 (0.74–4.15)	0.20	0.98 (0.57–1.68)	0.94
Aromatase inhibitors	1.47 (0.51–4.22)	0.48	0.90 (0.46–1.78)	0.77
Other, unknown type	1.99 (0.76–5.21)	0.16	0.74 (0.38–1.45)	0.38
Chemotherapy				
No	ref.		ref.	
Yes	1.40 (0.58–3.40)	0.45	1.09 (0.62–1.91)	0.76
Radiotherapy				
No	ref.		ref.	
Yes	1.49 (0.69–3.22)	0.31	0.88 (0.55–1.42)	0.60
Trastuzumab				
No	ref.		ref.	
Yes	0.36 (0.14–0.91)	0.03	0.50 (0.26–0.98)	0.04

Overall survival is defined as the absence of death (all causes). Breast cancer-specific survival is defined as the absence of (1) death due to breast cancer or (2) distant disease after which a person died. If a specific event occurred before start follow-up (breast cancer diagnosis or genetic testing, whichever came latest), this patient will be excluded from that specific analysis.

There were no proportional hazards violations, and therefore risks were estimated up to 10 years after BC diagnosis.

PYO=Person years of observation; HR = hazard ratio, 95%CI = 95 % confidence interval, ref = reference group.

### 3.2 Meta-analysis

We identified seven publications that studied one of the prognosis-related outcomes [7,[10], [11], [12], [13], [14]]. One study only describing recurrent disease-free survival was excluded [12], as this study also defined contralateral breast cancer as a recurrent disease (Supplementary Tables 7–8). Almost all studies showed a worse outcome compared to the non-carriers. Sample size of heterozygotes ranged from 34 to 963 within studies, all were tested in research setting for presence of *CHEK2* c.1100delC. Most studies compared the prognosis of *CHEK2* c.1100delC (also) with sporadic breast cancer patients, rather than with exclusively familial breast cancer patients. The majority of the patients were diagnosed before 2006. The majority of the studies included Dutch *CHEK2* c.1100delC breast cancer patients. As a result, there is a considerable overlap of *CHEK2* c.1100delC heterozygotes between studies.

For the meta-analysis, we aim to include patients only once. Due to the large overlap in heterozygotes between studies, we therefore selected only the data of the study with the largest number of heterozygotes for breast cancer-specific survival [5], in which all or the majority of the patients of the smaller studies were included [6,11].

After meta-analyzing these results with the results of our current study, a significant worse breast cancer-specific survival in *CHEK2* c.1100delC-associated breast cancer patients (RR = 1.29; 95 %CI = 1.06–1.58) remained compared to non-carriers. For overall survival, no significant difference was reported (Table 3).

**Table 3:** Meta-analyzed risk ratios and 95 % confidence intervals (95 %CI) for breast cancer-specific survival and overall survival based on results from previous studies and with additional inclusion of the current study.

Outcome	Risk ratio (95 %CI) of the selected published study^a^	Meta-analyzed risk ratios (95 %CI) of published study and the current study in a random effects model	I^2^ (%)	Meta-analyzed risk ratios (95 %CI) of published study and the current study in a fixed effects model	Weight of current study in the meta-analysis (%)
ER-positive patients					
Breast cancer-specific survival	1.38 (1.12–1.71)	1.11 (0.63–1.93)	69.2	**1.29 (1.06–1.58)**	11.1
Overall survival	1.43 (1.12–1.82)	**1.02 (0.50–2.08)**	86.7^¥^	1.22 (0.99–1.51)	15.5

As none of the previous studies reported on recurrent disease or distant disease in ER-positive breast cancer patients, no meta-analysis was performed and therefore not included in the Table.

In bold: the model that should be used based on the heterogeneity between the studies. ^¥^ P-value <0.05.

a As the same heterozygotes were included in multiple studies, a careful selection of studies was done to ensure that heterozygotes were included only once in the analysis. On breast cancer specific survival, there were three studies published, all using data from the Breast Cancer Association Consortium [[1], [2], [3]]. There was a lot of overlap in heterozygotes between the studies, therefore, we selected only the one with the largest number of heterozygotes [2]. For overall survival, there was only one study published [3]. More details on the studies can be found in the Supplements.

## 4 Discussion

Against our expectations based on the results from previous studies, *CHEK2* c.1100delC associated ER-positive breast cancer patients had similar recurrent disease-free survival, distant disease-free survival, breast cancer-specific survival and overall survival as compared to non-carriers. However, when meta-analyzing our results with previous studies, *CHEK2* c.1100delC was still associated with a worse breast cancer-specific survival.

When comparing the methodology of this study with previous studies on prognosis-specific outcomes for *CHEK2* c.1100delC heterozygotes, we did find some differences which may further explain why a worse prognosis was not found in our study.

First, we included patients diagnosed from 2006 onwards as this represents the more recent treatment regimens. From 2006 onwards, HER2-status of primary breast cancer is determined in diagnostic settings, allowing to study the impact of anti-HER2-treatment. This was not possible in studies with patients diagnosed in earlier years. While there is no evidence that *CHEK2* c.1100delC heterozygotes would respond better to targeted therapy, we found that heterozygotes are more often HER2-positive and therefore more often have been treated with targeted therapy. As the introduction of anti-HER2-treatment significantly improved the prognosis of HER2-positive breast cancer patients, we hypothesize that the worse survival described in previous studies might have been associated with HER2-positive breast cancer. With this more recent study, we found that *CHEK2* c.1100delC is significantly more often diagnosed with HER2-positive disease. Treatment with anti-HER2-treatment in *CHEK2* c.1100delC breast cancer patients could explain partially why no significant difference in prognosis was found anymore.

Second, as these patients were diagnosed in more recent years, the median follow-up time is shorter (6 years vs up to 10.9 years after breast cancer diagnosis in previous studies). Especially in studies including ER-positive breast cancer patients, a longer follow-up is crucial as the recurrence disease and metastatic disease often occur after stopping with endocrine therapy [23]. This was also shown in one study with a median follow-up of 7 years. This study included both ER-positive and ER-negative *CHEK2* breast cancer patients, and no significant difference in distant disease-free survival or breast cancer-specific survival for the first 6 years after diagnosis was found. However, after those 6 years, *CHEK2* c.1100delC was associated with a worse distant disease-free survival (HR = 2.7; 95 %CI = 1.8–3.9) and breast cancer-specific survival (HR = 2.1; 95 %CI = 1.4–3.0) [3]. In the current study, there was also a time-dependent change in risk estimates for recurrent disease and distant disease-free survival. However, after that, contrary to the previous study, we found a (non-significant) improved survival for the *CHEK2* c.1100delC patients compared to non-carriers. Therefore, we aim to extend follow-up in a future study, to validate our present findings.

Third, we compared the prognosis in *CHEK2* c.1100delC-associated breast cancer patients with the prognosis of patients who had an a-priori higher than average risk for breast cancer since they had an indication for genetic testing. All patients had to survive until the moment of testing (so-called “lead time”) [24], resulting in a survival bias for both groups. However, it is possible that this survival bias is different within each group. Non-carriers were selected from the hospitals’ databases, while *CHEK2* c.1100delC heterozygotes were invited to participate in the Hebon-CHEK2 study. Before sending out the invites, clinical geneticists were asked to provide input on whether or not women should be invited. Reasons not to invite also included when women were in the palliative phase of disease or situations in which receiving an invitation could cause more harm than good [20]. While only a few women were not invited in the Hebon-CHEK2 study for this reason, and the impact on the survival is expected to be small, there might be some residual bias present. As this study is only been conducted in a population of women who underwent genetic testing, further research is needed to understand whether these findings are generalizable for all breast cancer patients.

Also, as we only had information on vital status from non-responders, we were only able to study the overall survival. For the breast cancer-specific survival, we classified the deceased non-responders as breast cancer-specific deaths or as death by other causes. Furthermore, we once did the analyses without all non-responders. As we found similar results in the three separate analyses, we assumed that by removing the non-responders from the recurrent disease-free and distant disease-free survival analyses would not significantly impact our results. Furthermore, a recently published study on breast cancer patients with any *CHEK2* pathogenic variant showed a similar breast cancer-specific survival compared to the average genetically tested patients [25], which supports our current findings. Further research is needed to understand the potential impact of this bias.

Fourth, the definitions of outcomes are not always clearly described in the previous papers, especially regarding recurrent disease occurrences. Studies in our meta-analysis describing recurrent disease did not specify whether e.g. the occurrence of a second (contralateral) breast cancer diagnosis was defined as recurrent disease [7,8,12]. Increased risks of second (contralateral) breast cancer diagnosis have been described for *CHEK2* c.1100delC breast cancer patients [[2], [3], [4], [5],^11^], and could therefore have been a reason for the increased risk of recurrent disease reported previously. Based on current literature, second (contralateral) breast cancer is often considered as a new primary breast cancer diagnosis [26,27], rather than a recurrent disease. However, these results have influenced the medical knowledge on prognosis, and more specifically on recurrent disease, for *CHEK2* c.1100delC-associated breast cancer patients. Our current analysis is the first study on recurrent diseases and distant diseases specifically in ER-positive breast cancer patients, showing no worse outcome for *CHEK2* c.1100delC breast cancer patients compared to non-carriers.

Fifth, we found a large overlap in heterozygotes within the published studies. It might be possible that some of the patients included in the current study still overlap with patients from the previous studies. However, because of the different years of diagnosis and also because women in the current study were tested by a clinical genetics department rather than in research setting, we assume these chances are very small.

Although our results differ from previous studies in *CHEK2* c.1100delC breast cancer patients, one recent study in breast cancer patients with any (likely) pathogenic *CHEK2* variants also showed a similar breast cancer-specific survival in heterozygotes and non-carriers [25]. Furthermore, a recent review suggested that results from previous studies on survival in *CHEK2-*associated breast cancer patients are inconclusive when keeping the design of most studies into account, and stated that further prospective studies with longer follow-up periods are needed to study the survival [28]. Furthermore, this review suggests that all variables associated with survival should be considered, including treatment-related variables.

Strengths of this study include the large nationwide setting, reducing heterogeneity between patients and treatments given. This limits other types of bias that might have occurred in larger international studies, such as differences in treatment regimens and compliance between countries. It is also the first study that established risk estimates for recurrent disease-free survival and distant disease-free survival in ER-positive *CHEK2* c.1100delC breast cancer patients. As part of this nationwide study, we received nearly complete tumor data and follow-up for all responders, with only small numbers of missing values across the tumor characteristics and treatment. As a result, we were able to further stratify by HER2-status.

Limitations of this study include that the non-carriers were selected from two highly specialized cancer centers. We found significant differences in characteristics and treatment, such as tumor size, lymph node status, HER2-status, treatment with radiotherapy and types of endocrine therapy. Although we have adjusted for these factors, there might be some residual confounding. Second, we used 2015 as a cut-off for genetic testing, as *CHEK2* c.1100delC testing alongside *BRCA1* and *BRCA2* was implemented. Consequently, the majority of these women have not been tested for the whole *CHEK2* gene, a procedure which was implemented in 2018/2019, or any of the other breast cancer-associated genes that are usually tested nowadays (*ATM, BARD1, PALB2, RAD51C* and *RAD51C).* Some of the patients might still turn out to have a pathogenic variant in one of breast cancer associated genes which were more recently added to the breast cancer panel. Furthermore, ER-positive breast cancer patients are advised to undergo endocrine therapy for five years after breast cancer diagnosis. After that time, risk of recurrence increases steadily over time [23]. In this study, we focused on the 10-year risks and patients had a median follow-up of 6 years. For recurrent disease-free and distant disease-free survival, the curves diverted after 5 years. Although non-significant, we found a potential better survival for *CHEK2* heterozygotes after 5 years compared to the non-carriers in our study, which is contrary to previous results. Therefore, to further study the risk of recurrent disease and distant disease, a longer follow-up and more events are needed.

Concluding, unlike previous studies, we found no evidence for significant differences in prognosis in ER-positive *CHEK2* c.1100delC-associated breast cancer patients and non-carriers, which could be explained by the more recent years of diagnosis and treatment regimens. Meta-analyzing our results with older studies still showed a significant worse breast cancer-specific survival for heterozygotes. A potential explanation for this worse breast cancer-specific survival could be the large amount of missing data on HER2-status and the lack of targeted therapy in previous studies. Further research will shed light on these findings using nationwide selection of non-carriers consisting of both tested and unselected breast cancer patients with longer follow-up and more events.

## CRediT authorship contribution statement

**Maartje A.C. Schreurs:** Writing – review & editing, Writing – original draft, Formal analysis, Data curation. **Muriel A. Adank:** Writing – review & editing, Writing – original draft, Supervision, Funding acquisition, Data curation, Conceptualization. **Bernadette A.M. Heemskerk-Gerritsen:** Writing – review & editing, Writing – original draft, Data curation. **Antoinette Hollestelle:** Writing – review & editing, Writing – original draft, Supervision, Funding acquisition, Data curation, Conceptualization. **Nyrée Smallenbroek:** Writing – review & editing, Data curation. **Christi J. van Asperen:** Writing – review & editing, Data curation. **Margreet G.E.M. Ausems:** Writing – review & editing, Data curation. **Irma van de Beek:** Writing – review & editing, Data curation. **Geertruida H. de Bock:** Writing – review & editing, Data curation. **Ingrid Boere:** Writing – review & editing, Data curation. **Liselotte P. van Hest:** Writing – review & editing, Data curation. **Kim J.A.F. van Kaam:** Writing – review & editing, Data curation. **Linda de Munck:** Writing – review & editing, Data curation. **Janet R. Vos:** Writing – review & editing, Data curation. **Agnes Jager:** Writing – review & editing, Writing – original draft, Data curation. **Marjanka K. Schmidt:** Writing – review & editing, Writing – original draft, Supervision, Funding acquisition, Data curation, Conceptualization. **Maartje J. Hooning:** Writing – review & editing, Writing – original draft, Supervision, Funding acquisition, Data curation, Conceptualization.

## Ethics declaration

Hebon has a formal collaboration between all clinical genetics centers of the University medical centers and the Netherlands Cancer Institute. In addition, the updated Hebon study was approved by the institutional research board of the Netherlands Cancer Institute (19.221/IRBd19043) and medical ethical committee of the Maastricht UMC (11-4-089/2019-1388) and Erasmus MC (MEC-2011-471, amendment 2019). As part of the inclusion to the Hebon study, women signed an (online) informed consent.

## Funding

This project was funded by the 10.13039/501100004622Dutch Cancer Society, project number 10758.

## Declaration of competing interest

The authors declare that they have no known competing financial interests or personal relationships that could have appeared to influence the work reported in this paper.

## Data Availability

We have established a cohort of which multiple layers of data allow for integrative analysis. The Hebon study collaborators will continue to collect data and include women from families with an increased risk of breast cancer and/or ovary cancer. We encourage collaborations with researchers, who can apply for data by submitting a proposal to Hebon (hebon@nki.nl). All proposals will be reviewed on scientific quality and methodology by the Hebon steering committee.
