# Supplementary material for: Outcomes for ER-positive CHEK2 c.1100delC breast cancer patients compared with breast cancer patients without the variant
Source: Breast. 2025 Nov 24;85:104666. doi: 10.1016/j.breast.2025.104666 (PMC12702395; doi:10.1016/j.breast.2025.104666)
Supplement: Multimedia component 1 [file mmc1.docx]

|  | **Overall survival** | | **Breast cancer-specific survival** | |
| --- | --- | --- | --- | --- |
|  | heterozygotes | non-*CHEK2* | heterozygotes | non-*CHEK2* |
| Patients, n | 479 | 943 | 479 | 943 |
| Events, n | 30 | 74 | 19 | 39 |
| PYO | 1959 | 4061 | 1959 | 4061 |
| Incidence rate/1000 PYO | 15.3 | 18.2 | 9.7 | 9.6 |
|  | **HR (95%CI)** | **p-value** | **HR (95%CI)** | **p-value** |
| *CHEK2* c.1100delC status  Non-carriers  Heterozygotes | ref.  0.86 (0.57-1.31) | 0.50 | ref.  1.04 (0.61-1.79) | 0.88 |
| Age at diagnosis (per year increase) | 1.01 (0.99-1.03) | 0.22 | 0.98 (0.95-1.00) | 0.05 |
| Year at diagnosis (per year increase) | 0.90 (0.81-1.00) | 0.05 | 0.90 (0.78-1.03) | 0.12 |
| Tumor size  <2 cm  2-5 cm  >5 cm | ref.  1.68 (1.09-2.61)  4.36 (2.63-7.21) | 0.02  <0.001 | ref.  1.58 (0.89-2.82)  3.89 (1.97-7.69) | 0.12  <0.001 |
| Nodal status  Negative  Positive | ref.  2.44 (1.65-3.61) | <0.001 | ref.  4.72 (2.65-8.41) | <0.001 |
| Endocrine therapy  None  Tamoxifen  Aromatase inhibitors  Other, unknown type | ref.  1.15 (0.70-1.89)  1.46 (0.78-2.75)  0.85 (0.46-1.58) | 0.57  0.24  0.61 | ref.  2.17 (0.99-4.74)  2.17 (0.81-5.78)  2.16 (0.90-5.18) | 0.05  0.12  0.08 |
| Chemotherapy  No  Yes | ref.  1.47 (0.95-2.28) | 0.09 | ref.  3.27 (1.55-6.90) | <0.01 |
| Radiotherapy  No  Yes | ref.  1.12 (0.71-1.76) | 0.64 | ref.  1.84 (0.90-3.74) | 0.09 |
| Trastuzumab  No  Yes | ref.  0.53 (0.28-0.99) | 0.05 | ref.  0.52 (0.22-1.20) | 0.13 |

**Supplementary Table 1:** Univariable analyses of association between *CHEK2* c.1100delC and breast cancer-specific and overall survival (hazard ratio) in ER-positive breast cancer patients.

Overall survival is defined as the absence of death (all causes). Breast cancer-specific survival is defined as the absence of (1) death due to breast cancer or (2) distant disease after which a person died. If a specific event occurred before start follow-up (breast cancer diagnosis or genetic testing, whichever came latest), this patient will be excluded from that specific analysis.

There were no proportional hazards violations, and therefore risks were estimated up to 10 years after BC diagnosis.

PYO=Person years of observation; HR=hazard ratio, 95%CI=95% confidence interval, ref=reference group.

|  | **Distant disease-free survival** | | | | **Recurrent disease-free survival** | | | |
| --- | --- | --- | --- | --- | --- | --- | --- | --- |
|  | <5 years after breast cancer diagnosis | | <5 years after breast cancer diagnosis | | <5 years after breast cancer diagnosis | | <5 years after breast cancer diagnosis | |
|  | heterozygotes | non-*CHEK2* | heterozygotes | non-*CHEK2* | heterozygotes | non-*CHEK2* | heterozygotes | non-*CHEK2* |
| Patients, n | 272 | 819 | 161 | 371 | 252 | 761 | 146 | 343 |
| Events, n | 10 | 30 | 3 | 17 | 10 | 29 | 2 | 16 |
| PYO | 765 | 2245 | 249 | 561 | 693 | 1937 | 210 | 508 |
| Incidence rate/1000 PYO | 13.1 | 13.4 | 12.0 | 30.3 | 14.4 | 14.9 | 9.5 | 31.4 |
|  | **HR (95%CI)** | **p-value** | **HR (95%CI)** | **p-value** | **HR (95%CI)** | **p-value** | **HR (95%CI)** | **p-value** |
| *CHEK2* c.1100delC status  Non-carriers  Heterozygotes | ref.  0.97 (0.47-1.98) | 0.93 | ref.  0.39 (0.11-1.35) | 0.14 | ref.  1.02 (0.50-2.10) | 0.95 | ref.  0.31 (0.07-1.34) | 0.12 |
| Age at diagnosis (per year increase) | 0.98 (0.95-1.01) | 0.11 | 1.00 (0.96-1.04) | 0.97 | 0.97 (0.94-1.01) | 0.06 | 0.99 (0.95-1.04) | 0.73 |
| Year at diagnosis (per year increase) | 0.88 (0.73-1.05) | 0.16 | 1.02 (0.79-1.33) | 0.86 | 0.87 (0.73-1.05) | 0.15 | 1.18 (0.86-1.63) | 0.31 |
| Tumor size  <2 cm  2-5 cm  >5 cm | ref.  1.20 (0.59-2.43)  3.02 (1.32-6.89) | 0.61  <0.01 | ref.  1.83 (0.64-5.23)  7.31 (2.45-21.88) | 0.26  <0.001 | ref.  1.25 (0.61-2.55)  3.09 (1.34-7.12) | 0.54  <0.01 | ref.  1.47 (0.49-4.39)  4.08 (1.84-18.53) | 0.48  <0.01 |
| Nodal status  Negative  Positive | ref.  3.89 (2.05-7.39) | <0.001 | ref.  2.19 (0.90-5.30) | 0.08 | ref.  3.59 (1.88-6.85) | <0.001 | ref.  1.69 (0.67-4.27) | 0.27 |
| Endocrine therapy  None  Tamoxifen  Aromatase inhibitors  Other, unknown type | ref.  1.76 (0.71-4.37)  0.69 (0.14-3.41)  1.32 (0.49-3.60) | 0.22  0.65  0.58 | ref.  0.90 (0.28-2.89)  1.98 (0.56-7.02)  Non-informative | 0.86  0.29  - | ref.  1.57 (0.67-3.69)  0.60 (0.13-2.91)  0.82 (0.30-2.27) | 0.30  0.53  0.70 | ref.  1.17 (0.32-4.27)  2.28 (0.54-9.54)  Non-informative | 0.81  0.26  - |
| Chemotherapy  No  Yes | ref.  4.89 (1.74-13.75) | <0.01 | ref.  1.08 (0.41-2.82) | 0.88 | ref.  2.94 (1.24-7.03) | 0.02 | ref.  1.11 (0.39-3.11) | 0.85 |
| Radiotherapy  No  Yes | ref.  1.87 (0.78-4.45) | 0.16 | ref.  1.52 (0.45-5.19) | 0.50 | ref.  1.47 (0.64-3.32) | 0.36 | ref.  2.18 (0.50-9.48) | 0.30 |
| Trastuzumab  No  Yes | ref.  0.45 (0.16-1.26) | 0.13 | ref.  0.29 (0.04-2.16) | 0.23 | ref.  0.50 (0.18-1.40) | 0.18 | ref.  Non-informative | - |

**Supplementary Table 2:** Univariable analyses of association between *CHEK2* c.1100delC and distant disease-free survival and recurrent disease-free survival (hazard ratio) in ER-positive breast cancer patients.

Recurrent disease-free survival is defined as the absence of a loco-regional occurrence or distant disease. Distant disease-free survival is defined as the absence of distant disease.

Non-responders were excluded from the analysis on recurrent disease-free survival and distant disease-free survival, as no data on these outcomes were provided.

PYO=Person years of observation; HR=hazard ratio, 95%CI=95% confidence interval, ref=reference group.

|  | **Distant disease-free survival** | | | | | **Recurrent disease-free survival** | | | | | |  |
| --- | --- | --- | --- | --- | --- | --- | --- | --- | --- | --- | --- | --- |
|  | <5 years after breast cancer diagnosis | | ≥5 years after breast cancer diagnosis | | | <5 years after breast cancer diagnosis | | | ≥5 years after breast cancer diagnosis | | |  |
|  | heterozygotes | non-*CHEK2* | | heterozygotes | non-*CHEK2* | | heterozygotes | non-*CHEK2* | | heterozygotes | non-*CHEK2* | |
| Patients, n | 272 | 819 | | 161 | 371 | | 252 | 761 | | 146 | 343 | |
| Events, n | 10 | 30 | | 3 | 17 | | 10 | 29 | | 2 | 16 | |
| PYO | 765 | 2245 | | 249 | 561 | | 693 | 1937 | | 210 | 508 | |
| Incidence rate/1000 PYO | 13.1 | 13.4 | | 12.0 | 30.3 | | 14.4 | 14.9 | | 9.5 | 31.4 | |
|  | **HR (95%CI)** | **p-value** | | **HR (95%CI)** | **p-value** | | **HR (95%CI)** | **p-value** | | **HR (95%CI)** | **p-value** | |
| *CHEK2* c.1100delC status  Non-carriers  Heterozygotes | ref.  0.99 (0.44-2.21) | 0.98 | | ref.  0.39 (0.10-1.39) | 0.15 | | ref.  0.73 (0.35-1.53) | 0.41 | | ref.  0.29 (0.06-1.28) | 0.10 | |
| Age at diagnosis (per year increase) | 1.00 (0.96-1.03) | 0.82 | | 0.98 (0.94-1.03) | 0.52 | | 0.99 (0.96-1.02) | 0.61 | | 0.98 (0.93-1.03) | 0.41 | |
| Year at diagnosis (per year increase) | 0.90 (0.72-1.13) | 0.36 | | 0.96 (0.72-1.28) | 0.80 | | 0.99 (0.80-1.22) | 0.91 | | 1.00 (0.86-1.16) | 0.95 | |
| Tumor size  <2 cm  2-5 cm  >5 cm | ref.  0.87 (0.41-1.83)  1.61 (0.68-3.84) | 0.71  0.28 | | ref.  2.12 (0.66-6.74)  9.45 (2.66-33.57) | 0.20  0.001 | | ref.  0.93 (0.47-1.84)  1.83 (0.82-4.09) | 0.84  0.14 | | ref.  1.50 (0.64-3.52)  4.25 (1.45-12.49) | 0.56  <0.01 | |
| Nodal status  Negative  Positive | ref.  2.80 (1.39-5.63) | <0.01 | | ref.  2.35 (0.80-6.93) | 0.12 | | ref.  2.56 (1.36-4.85) | <0.01 | | ref.  2.32 (0.99-5.46) | 0.37 | |
| Endocrine therapy  None  Tamoxifen  Aromatase inhibitors  Other, unknown type | ref.  1.15 (0.44-3.04)  0.34 (0.07-1.76)  1.46 (0.45-4.78) | 0.78  0.20  0.53 | | ref.  0.59 (0.15-2.36)  1.12 (0.25-5.04)  Non-informative | 0.46  0.88  - | | ref.  0.94 (0.43-2.09)  0.23 (0.05-1.12)  0.66 (0.23-1.87) | 0.89  0.07  0.44 | | ref.  0.61 (0.22-1.69)  0.71 (0.21-2.42)  - | 0.81  0.32 | |
| Chemotherapy  No  Yes | ref.  3.52 (1.10-11.29) | 0.03 | | ref.  0.55 (0.14-2.07) | 0.37 | | ref.  2.12 (0.86-5.24) | 0.10 | | ref.  0.50 (0.14-1.73) | 0.27 | |
| Radiotherapy  No  Yes | ref.  1.30 (0.53-3.19) | 0.57 | | ref.  1.42 (0.41-4.95) | 0.59 | | ref.  0.79 (0.39-1.61) | 0.53 | | ref.  2.25 (0.52-9.86) | 0.28 | |
| Trastuzumab  No  Yes | ref.  0.31 (0.11-0.91) | 0.03 | | ref.  0.34 (0.04-2.74) | 0.31 | | ref.  0.36 (0.14-0.95) | 0.04 | | ref.  - | - | |

**Supplementary Table 3:** Multivariable analyses of association between *CHEK2* c.1100delC and distant disease-free survival and recurrent disease-free survival (hazard ratio) in ER-positive breast cancer patients.

Recurrent disease-free survival is defined as the absence of a loco-regional occurrence or distant disease. Distant disease-free survival is defined as the absence of distant disease.

Non-responders were excluded from the analysis on recurrent disease-free survival and distant disease-free survival, as no data on these outcomes were provided.

PYO=Person years of observation; HR=hazard ratio, 95%CI=95% confidence interval, ref=reference group.

**Supplemental Table 4:** Multivariable analyses of the association between the *CHEK2* c.1100delC variant and breast cancer-specific survival analyses (hazard ratio) in ER-positive breast cancer patients with different choices for coding of events in and exclusion of non-responders.

|  | **Breast cancer-specific survival**  *Deceased non-responders coded as BC-specific death* | | **Breast cancer-specific survival**  *Deceased non-responders coded as not BC-specific death* | | **Breast cancer-specific survival**  *All non-responders excluded* | |
| --- | --- | --- | --- | --- | --- | --- |
|  | heterozygotes | non-*CHEK2* | heterozygotes | non-*CHEK2* | heterozygotes | non-*CHEK2* |
| Patients, n | 479 | 943 | 479 | 943 | 363 | 943 |
| Events, n | 19 | 39 | 12 | 39 | 12 | 39 |
| PYO | 1959 | 4061 | 1959 | 4061 | 1508 | 4061 |
| Incidence rate/1000 PYO | 9.7 | 9.6 | 6.1 | 9.6 | 8.0 | 9.6 |
|  | **HR (95%CI)** | **p-value** | **HR (95%CI)** | **p-value** | **HR (95%CI)** | **p-value** |
| *CHEK2* c.1100delC status  Non-carriers  Heterozygotes | ref.  0.77 (0.42-1.39) | 0.38 | ref.  0.50 (0.25-1.00) | 0.05 | ref.  0.63 (0.32-1.25) | 0.19 |
| Age at diagnosis (per year increase) | 0.98 (0.96-1.01) | 0.28 | 0.98 (0.95-1.01) | 0.21 | 0.98 (0.95-1.01) | 0.20 |
| Year of diagnosis (per year increase) | 0.91 (0.78-1.06) | 0.23 | 0.86 (0.73-1.00) | 0.06 | 0.85 (0.73-0.99) | 0.04 |
| Tumor size  <2 cm  2-5 cm  >5 cm | ref.  1.05 (0.58-1.92)  1.95 (0.93-4.06) | 0.87  0.08 | ref.  1.20 (0.64-2.26)  2.12 (0.95-4.74) | 0.57  0.07 | ref.  1.19 (0.63-2.24)  2.22 (0.99-4.97) | 0.60  0.05 |
| Nodal status  Negative  Positive | ref.  3.65 (1.91-6.95) | <0.001 | ref.  3.18 (1.61-6.28) | 0.001 | ref.  3.23 (1.63-6.39) | 0.001 |
| Endocrine therapy  None  Tamoxifen  Aromatase inhibitors  Other, unknown type | ref.  1.75 (0.74-4.15)  1.47 (0.51-4.22)  1.99 (0.76-5.21) | 0.20  0.48  0.16 | ref.  1.73 (0.72-4.13)  1.49 (0.51-4.33)  1.06 (0.35-3.20) | 0.19  0.40  0.94 | ref.  1.58 (0.66-3.79)  1.35 (0.46-3.95)  1.39 (0.46-4.18) | 0.31  0.58  0.55 |
| Chemotherapy  No  Yes | ref.  1.40 (0.58-3.40) | 0.45 | ref.  1.40 (0.54-3.62) | 0.49 | ref.  1.43 (0.54-3.65) | 0.48 |
| Radiotherapy  No  Yes | ref.  1.49 (0.69-3.22) | 0.31 | ref.  1.43 (0.63-3.23) | 0.40 | ref.  1.46 (0.64-3.31) | 0.37 |
| Trastuzumab  No  Yes | ref.  0.36 (0.14-0.91) | 0.03 | ref.  0.35 (0.13-1.00) | 0.05 | ref.  0.35 (0.13-0.99) | 0.05 |

Breast cancer-specific survival is defined as the absence of (1) death due to breast cancer or (2) distant disease after which a person died.

There were no proportional hazards violations, and therefore risks were estimated up to 10 years after BC diagnosis.

PYO=Person years of observation; HR=hazard ratio, 95%CI=95% confidence interval, ref=reference group.

Deceased non-responders were aged 34, 35, 38, 41, 44, 64 and 84 at time of primary breast cancer diagnosis.

**Supplemental Table 5:** Multivariable analyses of association between *CHEK2* c.1100delC and overall survival, breast cancer-specific survival, distant disease-free survival and recurrent disease-free survival (hazard ratio) in ER-positive/HER2-negative breast cancer patients.

|  | **Overall survival** | | **Breast cancer-specific survival** | | **Distant disease-free survival** | | **Recurrent disease-free survival** | | |
| --- | --- | --- | --- | --- | --- | --- | --- | --- | --- |
|  | heterozygotes | non-*CHEK2* | heterozygotes | non-*CHEK2* | heterozygotes | non-*CHEK2* | heterozygotes | | non-*CHEK2* |
| Patients, n | 367 | 773 | 367 | 773 | 259 | 754 | 254 | | 749 |
| Events, n | 25 | 66 | 17 | 34 | 11 | 42 | 12 | | 40 |
| PYO | 1496 | 3350 | 1496 | 3350 | 805 | 2309 | 804 | | 2285 |
| Incidence rate/1000 PYO | 16.7 | 19.7 | 11.4 | 10.1 | 13.7 | 18.2 | 14.9 | | 17.5 |
|  | **HR (95%CI)** | **p-value** | **HR (95%CI)** | **p-value** | **HR (95%CI)** | **p-value** | **HR (95%CI)** | **p-value** | |
| *CHEK2* c.1100delC status  Non-carriers  Heterozygotes | ref.  0.63 (0.39-1.03) | 0.07 | ref.  0.77 (0.41-1.43) | 0.40 | ref.  0.64 (0.32-1.31) | 0.23 | ref.  0.57 (0.29-1.10) | 0.10 | |
| Age at diagnosis (per year increase) | 1.01 (0.99-1.03) | 0.47 | 0.97 (0.94-1.00) | 0.07 | 0.99 (0.96-1.02) | 0.62 | 0.99 (0.96-1.02) | 0.38 | |
| Year of diagnosis (per year increase) | 0.90 (0.79-1.01) | 0.08 | 0.86 (0.73-1.01) | 0.06 | 0.99 (0.82-1.19) | 0.88 | 1.04 (0.87-1.24) | 0.65 | |
| Tumor size  <2 cm  2-5 cm  >5 cm | ref.  1.20 (0.73-1.99)  3.47 (1.98-6.11) | 0.47  <0.001 | ref.  0.80 (0.41-1.56)  2.01 (0.95-4.28) | 0.52  0.07 | ref.  0.97 (0.50-1.91)  2.87 (1.42-5.84) | 0.94  <0.01 | ref.  1.00 (0.54-1.85)  2.63 (1.34-5.19) | 1.00  <0.01 | |
| Nodal status  Negative  Positive | ref.  2.41 (1.46-3.96) | 0.001 | ref.  3.75 (1.84-7.67) | <0.001 | ref.  2.38 (1.28-4.41) | <0.01 | ref.  1.99 (1.12-3.53) | 0.02 | |
| Endocrine therapy  None  Tamoxifen  Aromatase inhibitors  Other, unknown type | ref.  1.06 (0.60-1.89)  1.06 (0.52-2.18)  0.83 (0.41-1.71) | 0.84  0.87  0.62 | ref.  1.83 (0.73-4.62)  1.86 (0.61-5.65)  1.98 (0.69-5.68) | 0.20  0.28  0.20 | ref.  0.98 (0.45-2.14)  0.63 (0.22-1.84)  0.69 (0.25-1.91) | 0.95  0.40  0.48 | ref.  0.90 (0.45-1.78)  0.63 (0.24-1.66)  0.43 (0.16-1.12) | 0.75  0.35  0.08 | |
| Chemotherapy  No  Yes | ref.  0.98 (0.54-1.77) | 0.95 | ref.  1.30 (0.50-3.34) | 0.59 | ref.  1.84 (0.78-4.38) | 0.17 | ref.  1.44 (0.67-3.03) | 0.33 | |
| Radiotherapy  No  Yes | ref.  0.92 (0.55-1.54) | 0.75 | ref.  1.45 (0.64-3.30) | 0.38 | ref.  1.31 (0.61-2.83) | 0.49 | ref.  0.92 (0.49-1.72) | 0.78 | |

Recurrent disease-free survival is defined as the absence of a loco-regional occurrence or distant disease. Distant disease-free survival is defined as the absence of distant disease. Overall survival is defined as the absence of death (all causes). Breast cancer-specific survival is defined as the absence of (1) death due to breast cancer or (2) distant disease after which a person died. If a specific event occurred before start follow-up (breast cancer diagnosis or genetic testing, whichever came latest), this patient will be excluded from that specific analysis.

Non-responders were excluded from the analysis on recurrent disease-free survival and distant disease-free survival, as no data on these outcomes were provided.

There were no proportional hazards violations, and therefore risks were estimated up to 10 years after BC diagnosis.

PYO=Person years of observation; HR=hazard ratio, 95%CI=95% confidence interval, ref=reference group.

**Supplemental Table 6:** Multivariable analyses of association between *CHEK2* c.1100delC and recurrent disease-free survival, distant disease-free survival and survival (hazard ratio) in ER-positive/HER2-positive breast cancer patients.

|  | **Overall survival** | | **Distant disease-free survival** | | **Recurrent disease-free survival** | |
| --- | --- | --- | --- | --- | --- | --- |
|  | heterozygotes | non-*CHEK2* | heterozygotes | non-*CHEK2* | heterozygotes | non-*CHEK2* |
| Patients, n | 107 | 163 | 61 | 160 | 60 | 155 |
| Events, n | 5 | 7 | 2 | 5 | 1 | 5 |
| PYO | 441 | 700 | 198 | 487 | 196 | 482 |
| Incidence rate/1000 PYO | 11.3 | 10.0 | 10.1 | 10.3 | 5.1 | 10.4 |
|  | **HR (95%CI)** | **p-value** | **HR (95%CI)** | **p-value** | **HR (95%CI)** | **p-value** |
| *CHEK2* c.1100delC status  Non-carriers  Heterozygotes | ref.  0.97 (0.21-4.48) | 0.97 | ref.  0.34 (0.03-3.87) | 0.39 | ref.  0.25 (0.02-2.54) | 0.24 |
| Age at diagnosis (per year increase) | 1.07 (1.01-1.14) | 0.02 | 1.02 (0.93-1.13) | 0.61 | 0.99 (0.90-1.10) | 0.90 |
| Year of diagnosis (per year increase) | 1.11 (0.73-1.71) | 0.62 | 0.78 (0.41-1.49) | 0.46 | 1.03 (0.54-1.97) | 0.92 |
| Tumor size  <2 cm  2-5 cm  >5 cm | ref.  6.07 (1.37-26.89)  6.59 (0.54-80.69) | 0.02  0.14 | ref.  3.00 (0.38-23.75)  - | 0.30  - | ref.  1.48 (0.18-12.21)  non-informative | 0.71  - |
| Nodal status  Negative  Positive | ref.  1.47 (0.34-6.27) | 0.60 | ref.  5.27 (0.75-37.09) | 0.10 | ref.  3.76 (0.57-25.03) | 0.17 |
| Endocrine therapy  None  Tamoxifen  Aromatase inhibitors  Other, unknown type | ref.  0.75 (0.10-2.52)  0.09 (0.01-1.64)  0.23 (0.03-1.90) | 0.74  0.11  0.17 | ref.  non informative  -  non informative | -  -  - | ref.  non informative  -  non informative | -  -  - |
| Chemotherapy  No  Yes | ref.  24.58 (0.59-1029.06) | 0.09 | ref.  non informative | - | ref.  0.12 (0.00-9.10) | 0.34 |
| Radiotherapy  No  Yes | ref.  1.29 (0.24-5.27) | 0.88 | ref.  non informative | - | ref.  non-informative | - |
| Trastuzumab  No  Yes | ref.  0.01 (0.00-0.27) | <0.01 | ref.  non informative | - | ref.  - | - |

Recurrent disease-free survival is defined as the absence of a loco-regional occurrence or distant disease. Distant disease-free survival is defined as the absence of distant disease. Overall survival is defined as the absence of death (all causes). If a specific event occurred before start follow-up (breast cancer diagnosis or genetic testing, whichever came latest), this patient will be excluded from that specific analysis.

Breast cancer-specific survival could not be estimated for this population and was therefore not included in the Table.

Non-responders were excluded from the analysis on recurrent disease-free survival and distant disease-free survival, as no data on these outcomes were provided.

There were no proportional hazards violations, and therefore risks were estimated up to 10 years after BC diagnosis.

PYO=Person years of observation; HR=hazard ratio, 95%CI=95% confidence interval, ref=reference group.

**Supplementary Table 7:** Study characteristics of published studies on prognosis among *CHEK2* c.1100delC-associated breast cancer patients.

| **First author, year** | **Country, study** | **Population** | **Median follow-up (years)** | **Age at diagnosis** | **Year of diagnosis** | **Adjusted for left-truncation in analysis** | **Co-variates in analysis** |
| --- | --- | --- | --- | --- | --- | --- | --- |
| De Bock, 2004 [1] | The Netherlands, ORIGO | Non-*BRCA* families | 3.3 | 23-86 | 1996-2002 | No | - |
| De Bock, 2006 [2] | The Netherlands, ORIGO | Non-*BRCA* families | 3.3 | 23-86 | 1996-2002 | No | ER-status |
| Schmidt, 2007 [3] | The Netherlands, BOSOM | Unselected | 10.1 | <50 | 1970-1994 | Yes | Age and tumor characteristics |
| Weischer, 2012 [4] | BCAC | Familial and unselected | 6.6 | IQR: 43-63 | IQR: 1996-2004 | Yes | Age and year of diagnosis, BMI, menopausal status, tumor characteristics |
| Kriege, 2014 [5] | The Netherlands | Familial and unselected | 7.0 | 22-80 | >1970 | No, but reported no difference when left-truncating | Age and year of diagnosis, tumor characteristics and treatment |
| Morra, 2023a [6] | BCAC | Familial and unselected | 10.9 | IQR: 48-64 | 1942-2018 | Yes | Tumor characteristics and treatment |
| Morra, 2023b [7] | BCAC | Familial and unselected | 9.1 | IQR: 44-64 | IQR: 1996-2003 | Yes | Age and year of diagnosis, tumor characteristics and treatment |

BCAC=Breast Cancer Association Consortium; BOSOM=Breast cancer Outcome Study of Mutation carriers; ORIGO=Oorsprong van BoRstkanker InteGraal Onderzocht.

**Supplementary Table 8:** Overview of study results for all breast cancer patients and ER-positive breast cancer patients.

| First author, year | Number of heterozygotes | Number of non-*CHEK2* | Recurrent disease-free survival  Risk ratio (95%CI) | | Distant disease-free survival  Risk ratio (95%CI) | | Breast cancer-specific survival  Risk ratio (95%CI) | | | | Overall survival  Risk ratio (95%CI) | |
| --- | --- | --- | --- | --- | --- | --- | --- | --- | --- | --- | --- | --- |
| **All patients** | |  |  |  | |  | |  | |  | |  |
| De Bock, 2004 [1] | 34 | 102 | 3.86 (1.91-7.78)* | | 2.81 (1.20-6.58) | |  | | | | 1.76 (0.52-5.93) | |
| De Bock, 2006 [2] | 34 | 1,052 | 4.47 (1.88-10.60)* | |  | |  | | | |  | |
| Schmidt, 2007 [3] | 54 | 1,425 | 1.70 (1.20-2.40) | |  | | 1.40 (1.00-2.10) | | | |  | |
| Weischer, 2012 [4] | 459 | 25,112 |  | |  | | 1.64 (1.36-2.04) | | | | 1.47 (1.22-1.76) | |
| Kriege, 2014 [5] | 193 | 4,529 |  | | 1.31 (1.05-1.63)^¥^ | | 1.39 (1.08-1.79) ^¥^ | | | |  | |
| Morra, 2023a [6] | 561 | 33,702 |  | |  | | 1.43 (1.13-1.81) | |  | | 1.16 (0.96-1.39) | |
| Morra, 2023b [7] | 963 | 81,738 |  | |  | | 1.30 (1.09-1.56) | | | |  | |
| **ER-positive patients** | |  |  |  | |  | |  | |  | |  |
| Weischer, 2012 [4] | 290 | 14,234 |  | |  | | 1.63 (1.24-2.15) | | | | 1.43 (1.12-1.82) | |
| Morra, 2023a [6] | 408 | 22,089 |  | |  | | 1.55 (1.18-2.05) | | | |  | |
| Morra, 2023b [7] | 694 | 54,481 |  | |  | | 1.38 (1.12-1.71) | | | |  | |

* Study reported disease-free survival but used a different definition where contralateral breast cancer is included as a recurrent disease. Therefore, not included in the final meta-analysis. ^¥^ Study reported no difference in distant disease-free survival (HR=1.08; 95%CI=0.83-1.42) and breast cancer-specific survival (HR=1.00; 95%CI=0.71-1.41) within the first 6 years after diagnosis, after which the curves diverted (HR=2.65; 95%CI=1.79-3.93 for distant disease-free survival and HR=2.05; 95%CI=1.41-2.99 for breast cancer-specific survival). Therefore, the displayed HRs are based on archived results for the complete follow-up period.

**References**

1. de Bock, G.H., et al., *Tumour characteristics and prognosis of breast cancer patients carrying the germline CHEK2*1100delC variant.* J Med Genet, 2004. **41**(10): p. 731-5.

2. de Bock, G.H., et al., *Association between the CHEK2*1100delC germ line mutation and estrogen receptor status.* Int J Gynecol Cancer, 2006. **16 Suppl 2**: p. 552-5.

3. Schmidt, M.K., et al., *Breast cancer survival and tumor characteristics in premenopausal women carrying the CHEK2*1100delC germline mutation.* J Clin Oncol, 2007. **25**(1): p. 64-9.

4. Weischer, M., et al., *CHEK2*1100delC heterozygosity in women with breast cancer associated with early death, breast cancer-specific death, and increased risk of a second breast cancer.* J Clin Oncol, 2012. **30**(35): p. 4308-16.

5. Kriege, M., et al., *Survival and contralateral breast cancer in CHEK2 1100delC breast cancer patients: impact of adjuvant chemotherapy.* Br J Cancer, 2014. **111**(5): p. 1004-13.

6. Morra, A., et al., *The impact of coding germline variants on contralateral breast cancer risk and survival.* Am J Hum Genet, 2023. **110**(3): p. 475-486.

7. Morra, A., et al., *Association of the CHEK2 c.1100delC variant, radiotherapy, and systemic treatment with contralateral breast cancer risk and breast cancer-specific survival.* Cancer Med, 2023. **12**(15): p. 16142-16162.
